# Revealing the Genetic Diversity and Population Structure of Garlic Resource Cultivars and Screening of Core Cultivars Based on Specific Length Amplified Fragment Sequencing (SLAF-Seq)

**DOI:** 10.3390/genes15091135

**Published:** 2024-08-28

**Authors:** Jing Yang, Meile Sun, Xiangrong Ren, Pengbing Li, Jingtao Hui, Jun Zhang, Guocang Lin

**Affiliations:** Comprehensive Experimental Field, Xinjiang Academy of Agricultural Sciences, Urumqi 830000, China; yangjing@xaas.ac.cn (J.Y.); sunmeile@xaas.ac.cn (M.S.); renxiangrong@xaas.ac.cn (X.R.); lipengbing@xaas.ac.cn (P.L.); huijingtao@xaas.ac.cn (J.H.); zhangjun@xaas.ac.cn (J.Z.)

**Keywords:** garlic, SLAF-seq, genetic diversity, population structure, core material

## Abstract

Garlic is an important vegetable and condiment that has good medical and health care effects. At present, the origin of Chinese garlic and its association with other types of quality are limited to the molecular marker level, and there are few reports at the genome level. Therefore, this study is based on the specific length amplified fragment sequencing (SLAF-seq) of 102 copies of garlic germplasm resources, the group structure, and further screening of the core germplasm. SLAF-seq of 102 garlic cultivars yielded 1949.85 Mb of clean data and 526,432,275 SNPs. Through principal component analysis, evolutionary tree, population structure, and genetic relationship analysis, all garlic cultivars were divided into 3 groups. Among them, Group 1 contains 45 Chinese cultivars and 1 Egyptian cultivar, which are distributed mainly in the coastal and central areas of China. Group 2 contains 36 Chinese cultivars and 1 U.S. cultivar, which are distributed mainly in Northwest China. Group 3 contains 19 Chinese cultivars, which are distributed mainly in Xinjiang, China. The genetic diversity results indicate that the fixation index (Fst) values of Group 1 and Group 2 are lower than those of Group 1 and Group 3 and that the diversity of nucleotides (π) of Group 3 is greater than those of Group 2 and Group 1. Finally, the 30 parts of the cultivars were used as the core germplasms, and there was no difference between the two cultivars in terms of core quality. In summary, this study provides tags for the determination of garlic molecular markers and genotypes and provides a theoretical basis for subsequent resource protection and utilization, genetic positioning of important agronomic traits, and molecular marking agglomeration breeding.

## 1. Introduction

Garlic (*Allium sativum*) is a plant of the genus *Allium* in the Amaryllidaceae family of the order Asparagales and originates from parts of the Mediterranean, Central Asia, and Western Asia [1,2]. Garlic has been widely cultivated and used throughout history, but due to environmental and geographical changes, traces of this practice have gradually disappeared. Today, tracing the wild ancestors of garlic or even the specific area of domestication is impossible [3]. However, *Allium tuncelianum*, which grows wild in Turkey, shares clear similarities with garlic and is considered one of the candidate wild ancestors of garlic [4]. Garlic is an indispensable condiment in the daily lives of residents because of its pungent taste. Garlic has high nutritional value; is rich in polysaccharides; has antioxidant, bactericidal, immunomodulatory, and other activities; and has certain anti-cardiovascular disease, anti-diabetes, and anticancer effects through a variety of mechanisms [5]. Garlic has a vast planting area with rich and diverse germplasm resources in China. The garlic industry of China accounts for 74% of the total worldwide production [6]. However, owing to the large size of the garlic genome, studying garlic germplasm resources at the molecular level is difficult, and the application of molecular breeding technology in the garlic industry is very limited [1].

Molecular markers are convenient and effective genetic research tools that can be used to study the origin, inheritance, and trait differentiation of animals and plants, and they are highly important for the study of genetic variation in garlic populations [7]. To date, a variety of molecular markers have been identified in garlic. Random amplified polymorphic DNA (RAPD) markers were first used (1995) to study the origin of garlic. Among the 300 isozyme analysis cultivars, 48 were selected for RAPD analysis, which confirmed that the center of origin of garlic is in West Asia. The cultivars can be divided into four groups, and many garlic varieties are variants of *Allium longicuspis* [8]. In addition, correlations between the geographical distribution and genetic distance of garlic resources exist. Subsequently, RADP analysis was used to study the growth period of garlic and the mutation rate of regenerated callus seedlings [9,10]. Subsequently, amplified fragment length polymorphism (AFLP) markers were used for genetic diversity analysis and geographical origin analysis of garlic [11,12,13,14]. Compared with RAPD markers, AFLP markers are more accurate [11]. Furthermore, Ovesna et al. [15] used AFLP markers to evaluate the relationship between the S-alkyl cysteine sulfoxide (SACS) content and garlic genotype. Kamenetsky et al. [16] used AFLP markers to analyze group differences with respect to garlic flowering ability and organic sulfur content. Inter simple sequence repeat (ISSR) markers, simple sequence repeat (SSR) markers, and sequence-related amplified polymorphisms (SRAPs) have been used in garlic populations in recent years to study genetic diversity. SSR markers were used by Jabbes et al. and Son et al. [17,18] to study the genetic diversity of different garlic varieties and Allium species, respectively. Single nucleotide polymorphism (SNP) markers are also often used in genetic analysis and have been widely used in other plants. Due to the complexity of the garlic genome, the use of SNP markers in garlic developed late.

The genome of garlic has been successfully sequenced and published in China, providing a sequence basis for the development of garlic genomics and genotype analysis [1]. Unlike those of sexually reproduced crops, the morphological and genetic variation of garlic is greatly limited because of its highly sterile nature. Although the genetic diversity of garlic has been studied via morphological, physiological, isozyme, and molecular marker techniques, the exact population structure is still unknown, which limits an in-depth understanding of the evolutionary history of *Allium* crops. Currently, the widely accepted view is that garlic is divided into four groups and one subgroup, and the *A. longicuspis* group may be the basal group for the origin of cultivated garlic [4]. In recent years, the continuous advancement of sequencing technology has increased the demand for large-scale, high-throughput sequencing at low cost. This demand has promoted the rapid development of next-generation sequencing (NGS) technology [19]. The emergence of sequencing platforms such as Illumina MiSeq and HiSeq 2500, Roche 454 FLX Titanium, and Ion Torrent PGM has reduced the cost and increased the accuracy of sequencing covering the entire genome. NGS-based whole-genome-level genotyping technologies, such as whole-genome sequencing, resequencing, and simplified genome sequencing, have allowed the development of tens of thousands of SNP markers across the entire genome [19]. Compared with the use of traditional molecular markers, methods using SNP markers have the characteristics of high throughput, high efficiency, greater diversity, greater representativeness, greater practicality, and polymorphism [20]. Whole-genome sequencing (WGS) is a high-throughput sequencing technology that sequences the genomes of different individuals of species with known genome sequences and performs differential analysis on individuals or groups [21]. Large data analysis methods can provide more accurate analyses of the kinship between individuals and the structure of a group [22]. Garlic is grown mainly in Shandong, Henan, Jiangsu, Hebei, and Xinjiang in China. Currently, the origin of Chinese garlic and its relationship with other germplasms are limited to molecular marker analysis, and few reports exist at the genome level. Therefore, in this study, the entire genomes of 102 garlic germplasm resources (including 100 Chinese cultivars) were resequenced by resequencing technology; SNP sites were detected, screened, and typed; genetic diversity and population structure were analyzed; and core collections were screened. A comprehensive evaluation of the genetic diversity of Chinese garlic population germplasm resources will provide a molecular basis for the development and utilization of garlic germplasm resources, a theoretical basis for garlic origin and domestication research, and a foundation for the identification of functional genes for the development of garlic molecular markers.

## 2. Materials and Methods

### 2.1. Plant Material

A total of 102 garlic germplasm resources collected and preserved by the vegetable team of the Characteristic Horticultural Crops Research Laboratory of the Comprehensive Experimental Field of Xinjiang Academy of Agricultural Sciences were used as test cultivars, of which 100 were the main varieties in garlic production areas in China and 2 were collected from abroad (Table S1). All germplasm resources were planted at the vegetable base of the Comprehensive Experimental Field of the Xinjiang Academy of Agricultural Sciences (43°56′ N, 87°28′ E), which was cultivated via drip irrigation in an open field, and the front stubble was vacant land. One day before sowing, 1000 times the amount of liquid was prepared with 80% carbendazim wettable powder, the garlic seeds were soaked for 8~10 h, and the moisture content was controlled. The seeds were then placed in a cool, dry place, ready to be sown. The land was leveled before sowing, and the sowing time was late October of the previous year. Mechanical ditching, a ditch depth of 8~10 cm, a ditch spacing (row spacing) of 25 cm, artificial planting in the cultivation ditch, and a plant spacing of 8~10 cm were used. The other management practices were the same as the usual local field management practices. In April 2023, 1~2 cm^2^ young leaves from various garlic resources were collected for DNA extraction.

### 2.2. Preparation and Resequencing of DNA Libraries

In April 2023, 102 young leaves of garlic were collected and stored at −80 °C. The genomic DNA of each sample was extracted via the CTAB method. After the sample was ground, 800 μL of CTAB extraction buffer was added, and the mixture was placed in a water bath at 65 °C for 1 h and mixed by inversion every 15 min. A total of 800 μL of phenol/chloroform/isopropanol (25:24:1) was added, the mixture was mixed by inversion, the mixture was centrifuged at 12,000 rpm for 10 min, and the upper layer was transferred to a new 2 mL centrifuge tube. An equal volume of chloroform/isopropanol (24:1) was added, the mixture was mixed by inversion, the mixture was centrifuged at 12,000 rpm for 10 min, and the supernatant was transferred to a new 1.5 mL centrifuge tube. A volume of 0.6 times the volume of isopropanol was added, the mixture was allowed to precipitate at −20 °C for 1 h, the mixture was centrifuged at 12,000 rpm for 10 min, the supernatant was discarded, and the mixture was washed twice with 75% ethanol. The mixture was dried in a vacuum dryer for 40 min and then dissolved in water to obtain a DNA solution. The reference genome of garlic (Allium sativum: GCA_014155895.1) was predicted via electronic digestion using the restriction enzyme digestion combination of HaeIII + ScaI, and the sequence with a length of 464–494 bp was defined as the SLAF tag according to the principle of enzyme digestion scheme selection [1,23]. DNA fragments of the desired length (~500 bp) were recovered by electrophoresis via Covaris for random interruption, and an adapter was added for cluster preparation. Finally, sequencing was performed via the Illumina HiSeq^™^ 2000 platform.

### 2.3. Data Analysis

Raw data in Fastq format were first processed via fastp (version 0.23.4) software [24]. In this step, clean data were obtained by removing the adapter sequence from the original data, removing the reads with an N ratio greater than 5%, removing the low-quality reads (the base number of the quality value Q ≤ 7 accounts for more than 30% of the entire read), and calculating the Q30 and GC contents. All downstream analyses were based on high-quality, clean data. The clean data were aligned to the reference genome (Allium_sativum: GCA_014155895.1) of garlic via bwa-MEM2 (version: 2.2.1) software [25]. GATK (version: 3.8) and SAMtools (version: 1.9.1) were used to detect SNP variants, and the two results intersected [26,27].

### 2.4. Population Genetic Analysis

This analysis was based on SNP data from variant detection, filtered by the minor allele frequency (MAF: 0.05) and a deletion rate of less than 20% to obtain high-agreement SNPs. MEGA X software was used to construct a Kimura 2-parameter model via neighbor-joining (NJ), and the bootstrap was repeated 1000 times to construct a phylogenetic tree [28]. Based on the SNPs, admixture software was used to analyze the population structure of the research cultivars [29]. For the research population, the number of subpopulations (K value) was preset to 1–10 for clustering, the clustering results were cross-validated, and the optimal number of clusters was determined according to the trough of the cross-validation error rate. Q-matrix stacked plots for each K value were generated via the R language Pophelper package [30]. The smartpcr program in the EIGENSOFT software package was used to perform principal component analysis on the SNP data to obtain the clustering of samples. GCTA (version: 1.92.1) software was used to estimate the kinship between two individuals in a natural population [31].

### 2.5. Core Germplasm Screening

Core Hunter II can extract diverse, representative, and least redundant subsets from many germplasm resources to construct core germplasm or microcore germplasm. According to the genetic variant marker (SNP) data, combined with a variety of evaluation measures (i.e., modified Rogers distance and Shannon diversity index), the cultivars were weighted with high diversity, high representativeness, and high-level gene richness. Core Hunter II software was used to screen the total germplasm resources according to gradients of 0.1, 0.2, 0.3, 0.4, 0.5, 0.6, 0.7, 0.8, and 0.9 according to the weighted index modified Rogers distance (0.7) and Shannon diversity index (0.3) [32]. The gene coverage (CV) of the selected cultivars was evaluated, and the number of core germplasm cultivars was finally determined.

### 2.6. Analysis of Genetic Diversity in the Core Germplasm

This analysis was based on the following conventional genetic diversity indicators: observed heterozygosity, expected heterozygosity, Nei diversity index, Shannon–Wiener index, and polymorphism information content (PIC) for all germplasm cultivars and the selected core germplasm. The minor allele frequency (MAF) is the second most frequent allele at a locus in a population. If little or no difference exists between the germplasm resources and the core germplasm set screened in the MAF, the screening can be considered rational and accurate.

## 3. Results

### 3.1. Assessment of the Quality of the Resequencing Data from 102 Garlic Genomes

**Figure 1:**
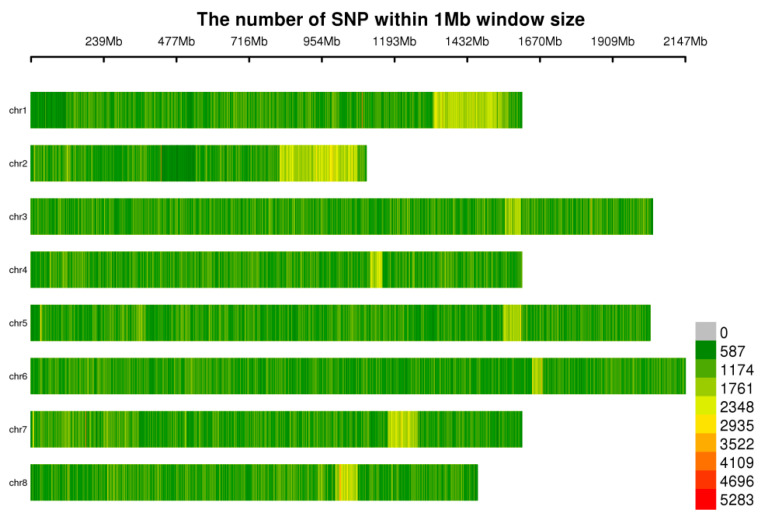
Distribution of SNPs on chromosomes. The abscissa is the length of the chromosome; each band represents a chromosome, and the genome is divided according to a size of 1 Mb. The more SNP markers present in each window, the darker red the color, and the fewer SNP markers present, the lighter green the color. Therefore, the darker red area in the figure is the area where the SNP markers are concentrated.

In this study, 102 garlic samples were analyzed via HaeIII + ScaI restriction enzyme digestion, and a total of 70,368,981 SLAF fragments (Table S3, Figure S1) were obtained. The SLAF fragments were subsequently sequenced, and a total of 1949.85 Mb of data were obtained after filtering. The sequencing depth of the samples was 5.37~35.86 X, the Q30 was 85.01~95.88%, the GC content was 38.47~43.31%, the effective number of reads in the reference genome was 95.35~99.80%, and 526,432,275 SNPs were obtained. After filtering by the minor allele frequency (MAF: 0.05) and a deletion rate less than 20%, 1,034,989 SNPs were finally obtained for subsequent analysis (Figure 1).

### 3.2. Phylogenetic and Principal Component Analyses of 102 Garlic Genomes

**Figure 2:**
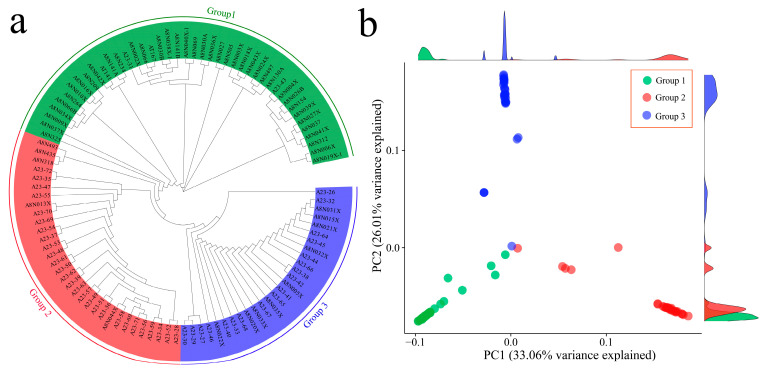
(**a**) Phylogenetic tree of 102 garlic samples; different colors represent different groups. (**b**) PCA of 102 garlic samples; different colors represent different groups.

Phylogenetic analysis of 102 garlic samples was performed via 1,034,989 SNPs, and the phylogenetic tree results revealed that all garlic cultivars could be divided into three groups (Figure 2a). The first group consisted of 46 submissions, the second group consisted of 37 submissions, and the third group consisted of 19 submissions. The figure shows that they are closest to each other. Furthermore, principal component analysis was performed on the SNPs of the samples, and the grouping information is marked in the scatter plot of the principal component analysis according to different colors (Figure 2b). The results of the principal component analysis were in good agreement with the results of the phylogenetic tree. PC1 could distinguish 102 garlic cultivars well, and a close relationship between Group 1 and Group 2 garlic germplasms was also observed.

**Figure 3:**
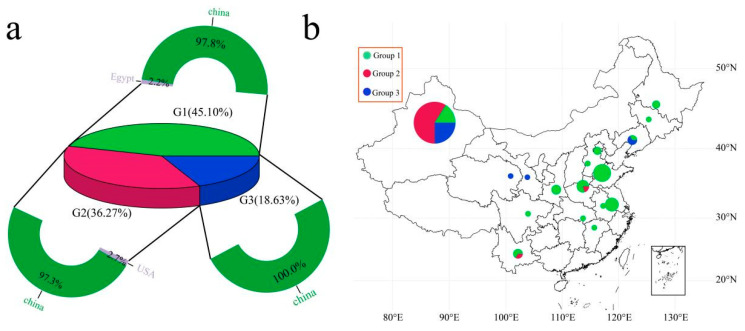
(**a**) Proportion of 102 servings of garlic ingredients; different colors represent different groups. (**b**) Geographical distribution of 100 Chinese garlic cultivars, with different colors representing different groups.

Through the phylogenetic tree of the samples and the PCA classification results, statistical analysis was performed on the three groups of samples (Figure 3a). Group 1 included 45 Chinese cultivars and 1 Egyptian cultivar, accounting for 45.10% of the total cultivars. Group 2 included 36 Chinese cultivars and 1 American cultivar, accounting for 36.27% of the total cultivars. Further analysis of the geographical distribution of 100 Chinese garlic cultivars revealed that Group 1 garlic cultivars were distributed mainly in coastal areas (mainly Shandong, Jiangsu, Henan, Shaanxi, Heilongjiang, Hebei, Anhui, Jilin, and Beijing), and Xinjiang in the northwestern inland region also contained 9 Group 1 cultivars (Figure 3b). Group 2 is mainly distributed in Xinjiang, China; Henan and Yunnan each contain one cultivar, and the remaining 34 are distributed in Xinjiang. Group 3 is distributed mainly in the northwestern inland region of China (Xinjiang, Gansu, Qinghai, and Liaoning), with Gansu and Qinghai each containing one cultivar, Liaoning containing two cultivars, and the remaining 15 cultivars being distributed in Xinjiang. According to the results of the phylogenetic tree and PCA, the relationships between Group 1 and Group 2 garlic germplasms were close, and the geographical locations of Xinjiang garlic and northwestern inland garlic were even closer.

### 3.3. Population Structure and Genetic Relationship Analyses of 102 Garlic Genomes

**Figure 4:**
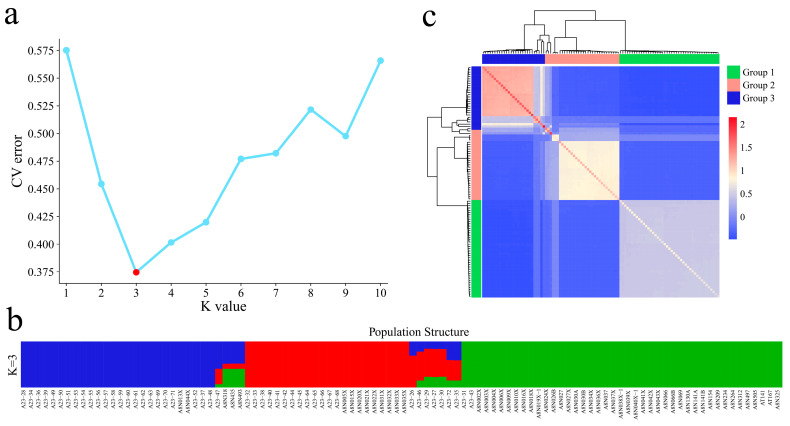
(**a**) Cross-validation error rate of 102 garlic samples with K values of 1–10; (**b**) clustering of 102 garlic samples with K = 3. (**c**) Cluster heatmaps of 102 garlic samples.

The coefficient of variation error (CV error) of the samples at different K values was calculated, and the coefficient of variation error rate was the lowest when K = 3 (Figure 4a); therefore, dividing the analyzed samples into three subgroups was most appropriate. According to the clustering results of the genetic structure data, all the samples were divided into three groups, namely, garlic from coastal areas of China, garlic from Xinjiang, and garlic from the northwestern inland region (Figure 4b). The geographical locations of Xinjiang garlic and northwestern inland garlic were more closely related, and the two groups of cultivars had highly consistent kinship results (Figure 4c). The garlic cultivars in Group 1 and Group 2 were mixed in Group 3, indicating that a wide range of garlic germplasm exchanges occurred in China. Moreover, Xinjiang cultivars contain garlic germplasm from other regions of China, and a single coastal and central garlic germplasm background is present, indicating that the coastal and central regions may be the centers of garlic in China.

### 3.4. Analysis of the Genetic Diversity between Groups

**Figure 5:**
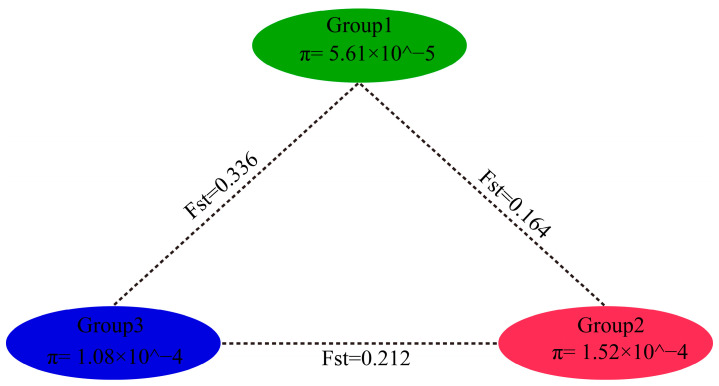
Nucleotide diversity and genetic differentiation coefficient between groups.

Genetic diversity refers to changes in genes within an organism, including genetic variation between distinct populations within a species and within the same population. Individuals within a population often do not have the same genotype, and the population is made up of multiple individuals with different genetic structures. The population fixation index (Fst) and π values among the different garlic sample groups were calculated to evaluate the degree of genetic differentiation between each garlic sample group and the genetic diversity within the group (Figure 5). The Fst values of Group 1 and Group 2 are lower than those of Group 1, Group 3, Group 2, and Group 3. This result is consistent with the results of phylogenetic, STRUCTURE, kinship, and principal component analyses; that is, the garlic germplasm of Group 2 was close to the genome of Group 1 and farther from that of Group 3. In terms of genetic diversity, the nucleotide diversity of Group 3 (π = 1.08 × 10^−4^) was greater than that of Group 2 (π = 1.52 × 10^−4^) and Group 1 (π = 5.61 × 10^−5^).

### 3.5. Core Material Screening

**Figure 6:**
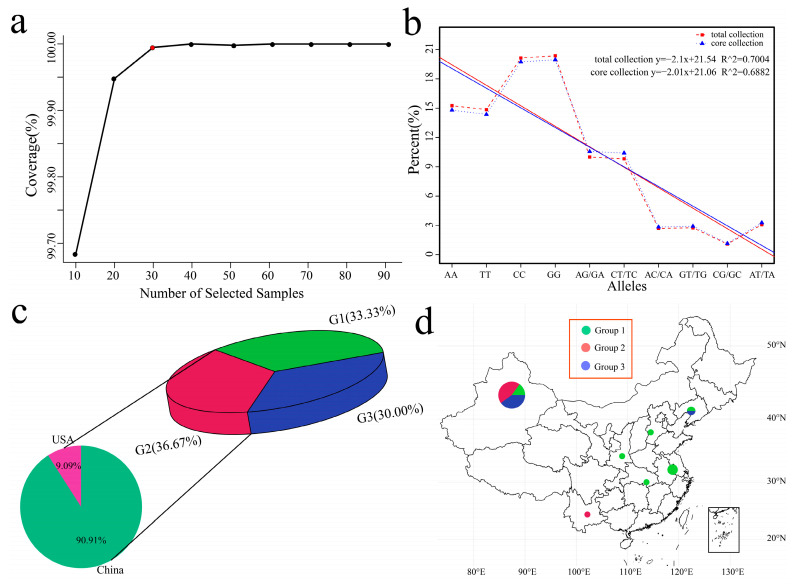
(**a**) Gene coverage (CV) evaluation. The abscissa represents the number of samples corresponding to the proportion of selected germplasm resources, the ordinate represents the CV value, and the red dots in the graph represent the candidate core germplasm. (**b**) Proportional distribution of genotypes, with the abscissa representing the ten genotypes; the ordinate represents the proportion of each genotype, the red dotted line represents the genotype distribution of all germplasm resources, and the blue dotted line represents the genotype distribution of the selected core germplasm. (**c**) Proportion of core garlic material; different colors represent different groups. (**d**) Geographical distribution of the core garlic material, with different colors representing different groupings.

The core germplasm represents the greatest genetic diversity of morphological characteristics, geographical distribution, genes, and genotypes of a species and its related wild species. Promoting germplasm exchange and utilization, as well as gene bank management, is highly practical. Core Hunter II can extract diverse, representative, and least redundant subsets from many germplasm resources to construct core germplasms. According to the genetic variant marker (SNP) data, combined with a variety of evaluation measures (such as modified Rogers distance and Shannon diversity index), the cultivars were weighted with high diversity, high representativeness, and high-level gene richness. Core Hunter II software was used to screen the total germplasm resources according to gradients of 0.1, 0.2, 0.3, 0.4, 0.5, 0.6, 0.7, 0.8, and 0.9 according to the weighted modified Rogers distance (0.7) and Shannon diversity index (0.3). The gene coverage (CV) of the selected cultivars was evaluated, and 30 cultivars were ultimately selected as the core germplasm (Figure 6a). Further evaluation of the observed heterozygosity, expected heterozygosity, Nei diversity index, Shannon–Wiener index, and polymorphism information content (PIC) of all the cultivars and core germplasms revealed that there was no significant difference between the two (Table S3). Based on the proportions of 10 genotypes among the 102 resource cultivars and 30 core germplasm cultivars, the frequencies of each genotype were close, and the fitted R2 was greater than 0.69, indicating that the 30 core germplasms we selected could replace the diversity of 102 resource cultivars (Figure 6b). Among the 30 core germplasms, Group 1 contained 10 Chinese cultivars, accounting for 33.33% of the 30 core germplasm cultivars (Figure 6c). Group 2 included 10 Chinese cultivars and 1 American cultivar, accounting for 36.67% of the total cultivars. Group 3 included 9 Chinese cultivars, accounting for 30.00% of the total cultivars. According to the geographical distribution of the 29 core germplasm cultivars in China, Group 1 was distributed mainly in coastal and central regions (mainly Hebei, Hubei, Jiangsu, and Liaoning), and Xinjiang in the northwestern inland region also contained 3 Group 1 cultivars (Figure 6d). Group 2 was distributed mainly in Xinjiang and Liaoning, and 8 cultivars were distributed in Xinjiang. Group 3 was distributed mainly in Xinjiang and Yunnan, with 9 shares.

## 4. Discussion

Genetic variation refers to the sum of the variation between different individuals or different species, which is essentially a change in the order of base pairs and the order of the structure of genetic material. The development of genetic diversity can help researchers intuitively understand the genetic structure and diversity of germplasm resources and provide a basis for breed differentiation, grouping, parental selection, and protection of wild and rare species in breeding work [33,34]. The study of genetic diversity through traditional morphological methods has the advantages of simplicity and intuitiveness, but the apparent morphology is affected by genes and the external environment, which can only indirectly reflect the essential characteristics of its genetic material. Moreover, the amount of detectable information is small, and certain defects and limitations exist [35]. Molecular marker technology is based on DNA polymorphisms, and the order of the DNA base double helix structure is the genetic information of crops; thus, direct analysis and comparison of the base sequence structure of DNA is the best way to analyze genetic diversity [36]. In the past decade, the rapid development of next-generation sequencing (NGS) and the continuous decline in sequencing costs have led to the rapid development of genomics in recent years, and an increasing number of animal and plant genomes have been assembled and published, laying the foundation for the identification of many SNPs in the whole genome of crops based on sequencing data [37,38]. Since the garlic genome is large and complex (16.24 Gb), it has many repetitive sequences (approximately 14.8 Gb, accounting for 91.3% of the garlic genome; 10.1% of the coding genes (5828) are distributed in the genome through tandem repeats) and high heterozygosity. SLAF-seq is a simplified genome sequencing technology that uses bioinformatics to find the most suitable enzyme to digest the genome, combined with a certain size of the insert fragment library, and high-throughput sequencing to quickly identify highly accurate variant marker (SNP) information in the whole genome. It is widely used in population evolution, functional gene positioning, molecular breeding, and germplasm resource identification. Compared with traditional sequencing technology, SLAF-seq is not limited by the reference genome and has the characteristics of a flexible enzyme digestion scheme, avoiding repetitive sequences, simplifying complex genomes, etc. The developed SNP molecular markers are cost-effective, stable, and evenly distributed in the genome, which can meet the analysis needs of large sample sizes. In this study, 102 garlic cultivars were identified via SLAF-seq, and phylogenetic tree construction, principal component analysis (PCA), genetic diversity analysis, and population structure analysis were carried out on the tested cultivars based on 1,034,989 high-quality SNP loci obtained via filtration. These variants represent large-scale, genome-wide variations in garlic and provide valuable resources for biological and breeding research in garlic.

In recent years, few reports on garlic germplasm resources have been published, which may be due to the large size of the garlic genome, which makes genome assembly slow. The sequencing of the garlic genome has laid a foundation for the construction of a garlic germplasm resource bank and the screening and cultivation of high-quality garlic varieties [1]. GBS was used to sequence 230 garlic germplasms to infer population structure, and four main groups of garlic germplasms were identified, among which the CG1 and CG2 groups contained germplasms of 46 cloves and multiple cloves of garlic in China, respectively [4]. In this study, 1,034,989 SNPs were used to analyze the phylogenetic and population structure of 102 garlic samples, and the phylogenetic tree results revealed that the Chinese garlic cultivars could be further divided into three groups. The same results were obtained for further PCA and population structure analyses. Group 1 is distributed mainly in coastal areas (mainly Shandong, Jiangsu, Henan, Shaanxi, Heilongjiang, Hebei, Anhui, Jilin, and Beijing), and Xinjiang in the northwest inland region also contains nine Group 1 cultivars. Group 2 is mainly distributed in Xinjiang, China; Henan and Yunnan each contain one cultivar, and the remaining 34 cultivars are distributed in Xinjiang. Group 3 is distributed mainly in the northwestern inland region of China (Xinjiang, Gansu, Qinghai, and Liaoning), with Gansu and Qinghai each containing one cultivar, Liaoning containing two cultivars, and the remaining 15 cultivars being distributed in Xinjiang. The germplasms of Group 1 and Group 2 garlic were closely related, the geographical locations of Xinjiang garlic and northwestern inland garlic were close, and the two groups of cultivars were still highly consistent in terms of kinship. The Xinjiang cultivars contain genotypes from garlic germplasms from other parts of China, and the coastal and central garlic germplasms of China have a single background, indicating that the coastal and central regions may be the origin centers of garlic in China. In terms of genetic diversity, the nucleotide diversity of Group 3 (π = 1.08 × 10^−4^) was greater than that of Group 2 (π = 1.52 × 10^−4^) and Group 1 (π = 5.61 × 10^−5^). This finding also indicates that the kinship of garlic species is affected mainly by geographical origin. The genetic proximity of cultivars from similar origins may be due to the tendency of the same breeding unit to use similar cultivars, resulting in a tendency for garlic varieties from some of the same regions to cluster together.

Recent advances in genome sequencing and molecular markers have provided a good way to study the genetic diversity of crops [39,40]. In recent years, genetic diversity analysis of important crops such as rice, *Zea mays*, and soybean by resequencing has revealed that cultivated crops may experience a decrease in genetic diversity in the process of artificial selection [41,42,43]. To better understand the genetic diversity of garlic germplasm resources in China, genetic diversity indices such as observed heterozygosity (Ho) and expected heterozygosity (He) were calculated. The heterozygosity of loci can reflect the genetic diversity of a population, whereas the inbreeding coefficient can be used as an indicator of the degree of inbreeding between individuals within a population. A Ho < He indicates that a certain degree of inbreeding exists within the analyzed population and that the species lacks sufficient heterozygotes, whereas a Ho > He indicates the opposite. Ho < He (0.291 < 0.341) of the Chinese garlic germplasm revealed that the genetic diversity within the population was low, which was conducive to the continuous evolution of this species. Fst is a statistical test used to measure the degree of differentiation between populations, and Fst ranges from 0 to 1; an Fst of 0 indicates that the genetic structure of different populations is completely consistent, and that no differentiation is present. An Fst of 1 indicates that the allele is fixed and fully differentiated in the population. The Fst range of garlic species from the CG1 and CG2 groups in China was 0.392 [4]. In this study, the Fst range between the three groups of Chinese garlic germplasms was 0.164~0.336, and the Fst between Group 1 and Group 3 was the largest (0.336), which was close to the value of 0.392 reported in a previous study. This further suggests that garlic in China can be divided into three subgroups. In conclusion, this study systematically analyzed the evolution and population structure of 102 garlic resource cultivars, provided comprehensive evidence for the prediction of garlic population differentiation, and provided a reference for the selection of appropriate resources for subsequent garlic germplasm breeding.

## 5. Conclusions

This study resequenced 102 garlic germplasm resources based on specific length amplified fragment sequencing (SLAF-seq) and constructed a whole-genome variation map containing 1.03 million SNPs. The 102 garlic resources were divided into three groups via high-quality SNP markers through principal component analysis, evolutionary analysis, population structure, and kinship analysis, and 30 materials were selected as core germplasms. These results have an important role in promoting the origin, evolution, and spread of Chinese garlic and in the protection of effective germplasm resources. This study provides a reference basis for the selection of basic resource material combinations in the subsequent breeding process of garlic, provides markers for the construction of fingerprint maps and the determination of genotypes, and provides a research basis for the subsequent protection and utilization of resources and the genetic positioning of important agronomic traits.

## Data Availability

The SLAF-seq data presented in the study are deposited in the NCBI repository under accession number PRJNA1120197.

## Supplementary Materials

The following supporting information can be downloaded at: https://www.mdpi.com/article/10.3390/genes15091135/s1, Table S1: Information on 102 garlic cultivars; Table S2: Statistical analysis of SLAF-seq data for all cultivars; Table S3: Genetic diversity assessment of all accessions and core collections; Figure S1: Distribution of SLAF fragments on chromosomes.
