# Supplementary figures and images for: Revealing the Genetic Diversity and Population Structure of Garlic Resource Cultivars and Screening of Core Cultivars Based on Specific Length Amplified Fragment Sequencing (SLAF-Seq)
Source: Genes (Basel). 2024 Aug 28;15(9):1135. doi: 10.3390/genes15091135 (PMC11431738; doi:10.3390/genes15091135)

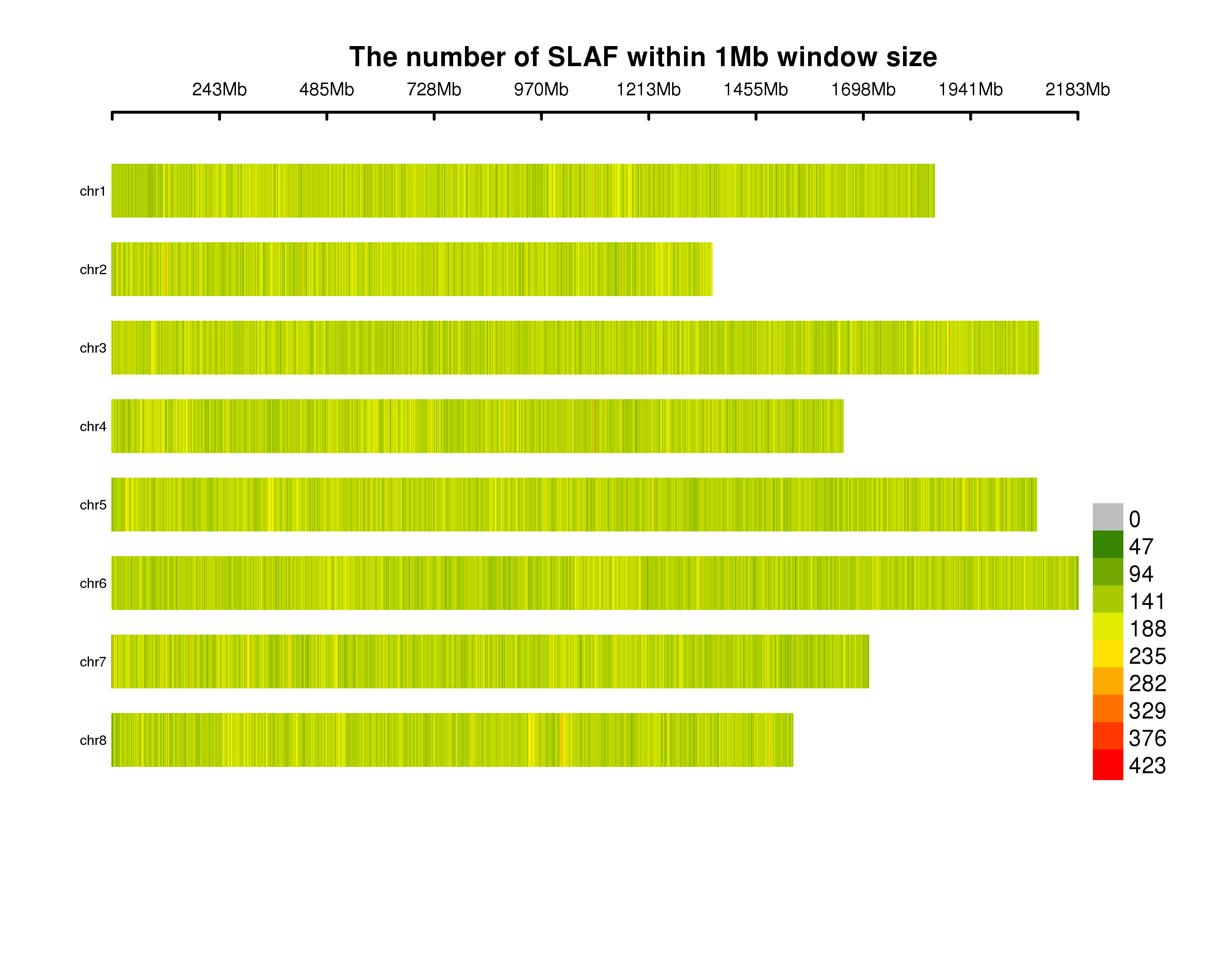

Supplement: Supplementary file 1 [file genes-15-01135-s001.zip › Supplementary Material/FIGURE S1.jpg]
